# ERP Responses to Regional Accent Reflect Two Distinct Processes of Perceptual Compensation

**DOI:** 10.3389/fnins.2019.00546

**Published:** 2019-06-14

**Authors:** Cesko C. Voeten, Clara C. Levelt

**Affiliations:** ^1^Leiden University Center for Linguistics, Leiden University, Leiden, Netherlands; ^2^Leiden Institute for Brain and Cognition, Leiden University, Leiden, Netherlands

**Keywords:** accent processing, perceptual compensation, language change, language variation, N400, P600

## Abstract

Humans possess a robust speech-perception apparatus that is able to cope with variation in spoken language. However, linguists have often claimed that this coping ability must be limited, since otherwise there is no way for such variation to lead to language change and regional accents. Previous research has shown that the presence or absence of perceptual compensation is indexed by the N400 and P600 components, where the N400 reflects the general awareness of accented speech input, and the P600 responds to phonological-rule violations. The present exploratory paper investigates the hypothesis that these same components are involved in the accommodation to sound change, and that their amplitudes reduce as a sound change becomes accepted by an individual. This is investigated on the basis of a vowel shift in Dutch that has occurred in the Netherlands but not in Flanders (the Dutch-speaking part of Belgium). Netherlandic and Flemish participants were presented auditorily with words containing either conservative or novel vowel realizations, plus two control conditions. Exploratory analyses found no significant differences in ERPs to these realizations, but did uncover two systematic differences. Over 9 months, the N400 response became less negative for both groups of participants, but this effect was significantly smaller for the Flemish participants, a finding in line with earlier results on accent processing. Additionally, in one control condition where a “novel” realization was produced based on vowel lengthening, which cannot be achieved by any rule of either Netherlandic or Flemish Dutch and changes the vowel's phonemic identity, a P600 was obtained in the Netherlandic participants, but not in the Flemish participants. This P600 corroborates a small number of other studies which found phonological P600s, and provides ERP validation of earlier behavioral results that adaptation to variation in speech is possible, until the variation crosses a phoneme boundary. The results of this exploratory study thus reveal two types of perceptual-compensation (dys)function: on-line accent processing, visible as N400 amplitude, and failure to recover from an ungrammatical realization that crosses a phoneme boundary, visible as a P600. These results provide further insight on how these two ERPs reflect the processing of variation.

## 1. Introduction

It has been successfully argued by many historical linguists that one of the key factors responsible for language variation and change, particularly when it relates to phonetics and phonology, is a poor ability of the human perceptual system to deal with unintentional variation in the speech signal, leading to *misperception* of a speaker by a listener (e.g.,Boyd-Bowman, 1954; Hyman, 1976, 2013; Ohala, 1981, 2012; Blevins, 2004; Bermúdez-Otero, 2015). However, multiple decades of research on speech processing by psycholinguists show that, in fact, the human speech system is very capable of handling non-meaningful variation, such as variation due to anatomical differences between speakers or the use of a regional accent. Processes such as perceptual learning (Norris et al., 2003), rate normalization (Bosker and Reinisch, 2015), compensation for coarticulation (Mann and Repp, 1980), and many other innate or acquired perceptual skills (cf.Cutler, 2012) enable the listener to accurately make the link between the forms speakers intend vs. the sounds they actually produce. If historical linguists are correct that the driving force behind linguistic change (and particularly sound change) is misperception, then the question is when and how these perceptual-compensation processes found in psycholinguistics “give way”, i.e. fail to correctly compensate for variation, thus enabling historical sound change to actuate. In empirical terms: under what conditions can we detect psycho- or neurolinguistic correlates of *unsuccessful* perceptual compensation for variation? The present paper provides a starting point in answering this question using evidence from ERP data.

Evans and Iverson (2007) have shown that it is possible to detect long-term accommodation to variation in speech, by investigating the speech production and perception of 19 English first-year university students. These students hailed from different dialect regions of the United Kingdom, and were shown behaviorally to adapt their speech production to the Standard-Southern-British-English university norms. In addition, a correlation was found with participants' perception of accented speech, but the latter did not reliably change on its own over time. The present study takes a similar approach, but focuses on the processing aspect. The language used for the investigation is Dutch. Dutch is spoken both in the Netherlands and in the northern part of Belgium (henceforth: Flanders), but due to thorough standardization processes that took place in the Netherlands but not in Flanders (Grondelaers and van Hout, 2011), there are significant differences in the phonological systems of these two varieties. The Netherlandic variety (henceforth: Standard Dutch), has undergone changes in its distribution of the tense mid vowels (/e:,ø:,o:/), diphthongs (/ɛi,œy,ɑu/), and rhotic (/r/). Specifically, Standard Dutch has diphthongal realizations of /e:,ø:,o:,ɛi,œy,ɑu/ (thus realizing these vowels as [ei,øy,ou,ɛi,œy,ɑu]) and a glided coda /r/ (realized [ɹ]), whereas the Belgian variety (henceforth “Flemish Dutch”) has monophthongal realizations of /e:,ø:,o:/ (yielding realizations [e:,ø:,o:]), markedly less diphthongization in /ɛi,œy,ɑu/, and does not glide the coda rhotic (thus realizing it [r]). These differences have all arisen via sound changes that have taken place in Standard Dutch but not in Flemish Dutch (Van de Velde, 1996; Sebregts, 2015). This makes the present-day variation between Standard Dutch and Flemish Dutch a useful proxy for historical sound change.

The study reported here investigates the perception of these speech sounds in speakers of Flemish Dutch who have migrated to the Netherlands. Ten Flemish-Dutch speakers (henceforth: FDS), all first-year university students who migrated to the Netherlands, are compared to 10 Standard-Dutch speakers (henceforth: SDS). Participants are tested multiple times to test for possible longitudinal adaptation on the part of the Flemish students. Using an exploratory extension of the violation paradigm (Friederici et al., 1993) to phonological processing, the objective of the investigation is to find behavioral and electrophysiological correlates of the processing of the type of variation under discussion. While it will turn out that this endeavor will be unsuccessful, two robust differences between the two groups provide new information about the types of phonological variation whose processing neurolinguistic methods can detect. It will be shown that the FDS are less able to “take in” SDS speech, which is reflected by a smaller N400 decrease over two repetitions of the same experiment compared to the SDS (cf. behavioral findings by Floccia et al., 2009). In addition, it will be shown that there are ERP-detectible differences in the processing of SDS speech between the two groups. Specifically, in words where the vowel /ɛ/ is realized as [ɛ:]—an ungrammatical realization that cannot be achieved by any known phonological rule of Standard Dutch—a P600 is obtained in the SDS, but not in the FDS. This is in line with previous research (Pater et al., submitted; Domahs et al., 2009) about the role of the P600 in the processing of phonological rules.

The remainder of the paper is structured as follows. Section 2 discusses the psycholinguistic and neurolinguistic correlates of accent (violation) processing that have been identified in the prior literature. Due attention is paid to the well-known N400 component, and to the P600 which is a relatively new, but not unknown, component in this field. Section 3 details the methodology used in the present experiment. Section 4 provides the results, which are discussed in section 5, first separately for the two findings (N400 and P600) and then together. Finally, section 6 concludes the paper.

## 2. Accent Processing

At its core, the present study is about accent accommodation, a subfield intersecting psycholinguistics and phonetics. Previous research in this field has shown that listeners are very adept at compensating for linguistic variation, particularly in the vowel system. Maye et al. (2008), for instance, show that listeners are able to accommodate to a completely novel vowel shift in English (all vowels lowered by one degree, so “wicked witch” becomes “weckud wetch”) after only a few minutes of exposure. At the same time, however, Floccia et al. (2006) found that a notable regional accent incurs a slowdown in lexical decision tasks of about 30 ms. This effect accumulated over time, i.e., with longer words this delay increased more than proportionally. This suggests an interference effect starting from the very beginning of lexical processing, which persists even as the listener receives more exposure to the accented speech (Floccia et al., 2009).

These results suggest that while participants in behavioral tasks are able to accommodate successfully in order to fulfill the task, their processing is still somehow impaired when confronted with accentual variation. This processing difficulty has been measured directly in ERP investigations. Goslin et al. (2012) found that accented realizations reduced the amplitude of both the phonological-mapping negativity (otherwise known as the N280) and the N400. The relationship between accent and the phonological-mapping negativity is obvious, but the involvement of the N400 might be considered surprising, given that this component is normally connected to semantic processing, or more generally to lexical predictions (Dambacher et al., 2006). The results to be presented in section 4 will give reason to postulate two different ways in which the N400 may be modulated by accentual variation. On the one hand, the findings by Goslin et al. (2012) suggest that a persistent regional accent reduces the strength of the lexical predictions made by a listener, resulting in a *reduced* overall N400 amplitude due to simple parsing difficulty, which causes the listener to predict more cautiously. On the other hand, the results from the present paper will give reason to postulate an N400-*increasing* effect for regionally-accented words, due to their decreased consolidation in the lexicon over time.

Recent studies have intimated another ERP component in accented-speech processing: the P600. Originally known from syntax (Osterhout and Holcomb, 1992), it was observed by Liu et al. (2011) that a P600 could also be elicited by phonology. Specifically, Liu et al. (2011) observe a P600 in Chinese participants who read well-known poems in which some words were replaced with synonyms that only differed in orthographical and phonological form. They argue that the P600 that was elicited by these “deviant” words must be due to phonological processing, since other sources of integration difficulties such as semantic violations were absent. Kung et al. (2014) found a similar effect in a lexical-decision task in Chinese. In this task, Chinese words with a low lexical tone were embedded as the final word in a sentence that carried the intonation pattern of a question. Because questions in Chinese end with rising intonation, the pitch contour of such words is very similar to the pitch contour of words with a high lexical tone in a regular statement sentence. The resulting processing difficulty (“is this a word with a low lexical tone that rises because the sentence is a question, or is the sentence a statement and does the word simply carry a high lexical tone?”) manifested as a P600, which Kung et al. (2014) interpret as being caused by reanalysis when the listener resolves this conflict by choosing (in these cases) for the question interpretation.

Phonological P600 effects have also been found beyond prosody, viz. in the domain of segmental phonology. Domahs et al. (2009) obtained a P600 in a lexical-decision task, and Pater et al. (submitted) found a P600 in a phonological artificial-language-learning task. Both of these studies investigated a specific subset of accented speech, viz. violations of allophonic rules: Pater et al. (submitted) violated an artificially-learned voicing-agreement rule, and the Domahs et al. (2009) study found a P600 when two stop consonants followed each other in a way that violated the phonotactics of German (the native language of their participants). It is important to mention that both violations were neutralizing: the violating consonant was already present on its own in the phoneme inventory. Behavioral findings by Witteman et al. (2015) suggest that this matters: they show that adaptation to an accent in general is possible, but not to individual sounds that cross a phoneme boundary. Furthermore, there is more interference when a single sound in a word is replaced by a realization *that does not occur normally* (e.g., Dutch /œy/ replaced by German [ɔɪ], which does not exist in Dutch; Witteman et al., 2014).

It thus appears that, even though adaptation is possible, there are multiple behavioral and electrophysiological correlates of problems faced by listeners when processing accented speech. In reaction-time experiments, they are slower. In ERP studies, the N280, N400, and P600 play a role. Two of the four phonological P600 studies discussed found the effect in the context of allophonic-rule violations. The present study integrates these results and attempts to extend them by investigating reaction times and ERP responses to Standard Dutch speech by Flemish Dutch students. The approach is similar to that taken by Witteman et al. (2015) and used in the P600 studies by Domahs et al. (2009) and Pater et al. (submitted): only a single sound is manipulated in an otherwise normal Standard Dutch word.

Given the above, the aim of the present study is to investigate two things. Behaviorally, it can be expected that the well-known effect of *identity priming* (participants being faster to read a word aloud if they have just been presented the same word auditorily) will be less strong for realizations that do not conform to participants' phonological grammars. Specifically, the expectation is that unmanipulated identity primes facilitate word reading, but manipulated identity primes incur the same RT slowdown reported by Floccia et al. (2006, 2009) on top of this identity-priming effect. Electrophysiologically, the manipulations are expected to specifically elicit a P600 ERP when they result in ungrammatical allophones, and possible across-the-board N280 and N400 effects may arise in general.

The hypotheses presented above are evaluated for Standard-Dutch speakers (henceforth: SDS) and Flemish-Dutch speakers (henceforth: FDS), who have only just moved to the Netherlands to start their university studies there (parallelling Evans and Iverson's 2007 study). The expectation is that there will be differences, but that they will reduce over months of time as the FDS participant receive more exposure; the present experiment takes 9 months divided into three sessions. Differences are expected in terms of RTs and in terms of ERPs. Concerning RTs, the expectation is that the hypothesized difference in identity-priming effects will be smaller for the FDS than for the SDS, as the FDS will have remaining difficulty parsing also the non-manipulated segments of the words. In terms of ERPs, it is expected that the FDS have different N400 responses (regardless of task or type of violation), in line with findings by Goslin et al. (2012). In addition, the P600 effect in response to allophonic violations (Pater et al., submitted; Domahs et al., 2009) is expected to be smaller for the FDS than for the SDS, as the FDS should have less robust prior expectations due to having had less exposure to Standard Dutch speech.

The task used in the present study, explained in section 3, has not been used in a violation paradigm before, and the research is therefore of an exploratory nature. The results will show that the task is sensitive to phonological violations in individual speech sounds, but that this effect must have a deeper source than surface allophones: it will be shown that the task cannot detect contextual violations, but is instead sensitive only to realizations that lie outside the set of possible realizations of a phoneme, further precising the behavioral findings by Witteman et al. (2014, 2015).

## 3. Materials and Methods

### 3.1. Participants

Participants consisted of 10 FDS participants (seven female, three left-handed; mean age = 22.71 years, SD = 3.54 years) and 10 SDS controls (seven female, one left-handed; mean age = 20.53 years, SD = 2.48 years). The FDS participants were all in their first year of study at a Dutch university in the Randstad (either Leiden University or the University of Amsterdam) and were speakers of a variety of Flemish Dutch. The control participants were Standard-Dutch students, not necessarily in their first year, who were also studying in the Randstad and had grown up in a Randstad-Dutch environment. The FDS participants were tested as soon as possible after the beginning of the academic year (mean number of days past September 1st = 21.5 days; SD = 7.93 days).This restriction was not applied to the control group (mean number of days past September 1st = 104.30 days; SD = 54.40 days).

To find out about possible longitudinal adaptation processes, participants were tested over the course of three sessions. The mean interval between the sessions was 129.29 days (SD = 23.19) for session 1–2, and 112.75 days (SD = 22.94) for session 2–3. The experimental procedure and tasks, which are described below, were the same for all three sessions. In the end, 23 FDS datasets were collected and 28 SDS datasets; the discrepancy with the expected 3 × 10 = 30 datasets per group is due to drop-outs (session 1–2: two left-handed FDS and one right-handed SDS; session 2–3: one additional right-handed FDS) and equipment failure (one left-handed FDS in session 1 and one right-handed FDS in session 2). Table 1 lists the final set of participants present in the sample.

**Table 1:** Overview of the final population from which data were obtained.

	**Session**		
**Participant**	**1**	**2**	**3**
FDS-0		✓	✓
FDS-1	✓	✓	
FDS-2	✓		
FDS-3	✓	✓	✓
FDS-4	✓	✓	✓
FDS-5	✓	✓	✓
FDS-6	✓	✓	✓
FDS-7	✓		
FDS-8	✓	✓	
FDS-9	✓	✓	✓
SDS-0	✓	✓	✓
SDS-1	✓	✓	✓
SDS-2	✓	✓	✓
SDS-3	✓	✓	✓
SDS-4	✓	✓	✓
SDS-5	✓	✓	✓
SDS-6	✓	✓	✓
SDS-7	✓		
SDS-8	✓	✓	✓
SDS-9	✓	✓	✓

### 3.2. Stimuli

A phonetically-trained female speaker from the Randstad area of the Netherlands produced 309 prime words embedded in a carrier sentence. The experiment was an exploratory part of a larger battery of both neurolinguistic and non-neurolinguistic tests; for purposes of the present paper, 160 of these words are relevant and the remainder are fillers. The 160 experimental words comprised 8 groups of 20 words containing one of the phonemes /e:,ø:,o:,ɛi,œy,ɑu,a:ʀ,ɛ/ (the last vowel does not differ between Standard Dutch and Flemish Dutch and was included as a control) in stressed^1^ position. An equal number of fillers was used, containing the same phonemes separated into the same conditions, but positioned in a different phonotactic environment, namely preceding coda /l/. In this environment, the Standard-Dutch realization of these vowels is the same as the Flemish-Dutch realization. An obvious exception was made for consonantal control /a:ʀ/, which cannot be followed by coda /l/; this condition was simply included as a target twice, in order to retain the balance of the conditions presented to the participants. The same held for the /ɑul/ condition, as a lexical gap in Dutch prevents the vowel /ɑu/ from being followed by coda /l/. For reasons of convenience, 3 × 3 words beginning with one of the point vowel phonemes /i,u,a:/ were also included as fillers, both before /l/ and before non-/l/.

The 309 prime words thus present in the design were selected on the basis of frequency: for each cell, the 20 words selected are the 20 most-frequent words according to CELEX (Baayen et al., 1995) starting with the relevant phoneme(s) (mean log frequency = 6.41; SD = 2.07). The critical phonemes were always located at the beginning of words to maximize any possible priming effects on participants' reaction times, and to enable time-locking of ERPs to the onset of the critical manipulations. There were two exceptions: the requirement of word-initiality was dropped for the vowel /ø:/, as no words beginning with stressed /ø:/ were available in the corpus, presumably as a result of this vowel's general low frequency in Dutch. The initiality requirement was also rescinded for the filler items.

All primes, both target and filler with the exception of the nine point-vowel tokens, were recorded in two different variants. One of these variants was typical for SDS phonology and one of these variants was atypical of SDS phonology (and, in all non-filler non-control items, typical of FDS phonology). These two variants will henceforth be referred to as “SDS realizations” and “non-SDS realizations.” Table 2 summarizes the design. For the SDS realizations, the vowels /e:,ø:,o:,ɛi,œy,ɑu/ were realized as [ei,øy,ou,ɛi,œy,ɑu] in the experimental items. This is typical of SDS phonology, but atypical for FDS phonology. For the filler items where these vowels were followed by a coda /l/, they were realized as [e:,ø:,o:,ɛ:,ø:,ɑ:], which is typical for both SDS and FDS phonology. For the /ɛ/ control, the vowel was realized as [ɛ] (typical for both SDS and FDS), and for the /a:ʀ/ control, the sequence was realized [a:ɹ] (typical of SDS only). For the non-SDS realizations of the experimental items, the vowels /e:,ø:,o:,ɛi,œy,ɑu/ were realized as [e:,ø:,o:,ɛ:,ø:,ɑ:], which is typical of FDS but not permissible in SDS given the lack of a following coda /l/ in the experimental items. For the filler items where these vowels were followed by a coda /l/, they were realized as [ei,øy,ou,ɛi,œy,ɑu], which is not grammatical in FDS (where these realizations simply do not occur) or SDS (because these realizations are not permitted before coda /l/). The non-SDS /ɛ/ control was realized as [ɛ:], which is a phonologically illicit realization in either SDS or FDS speech, and the /a:ʀ/ control was realized as [a:ʀ], which does not apply the SDS-typical but FDS-atypical rule gliding /ʀ/ to [ɹ] in coda position.

**Table 2:** Overview of the allophone variants used in the experimental items.

	**Realization (SDS vs. Non-SDS) used in prime items**							
	**Before non-/l/ (Target)**				**Before /l/ (Filler)**			
**Phoneme**	**SDS**	**Typical for**	**Non-SDS**	**Typical for**	**SDS**	**Typical for**	**Non-SDS**	**Typical for**
e:	ei	SDS	e:	FDS	e:	FDS	ei	Neither
ø:	øy	SDS	ø:	FDS	ø:	FDS	øy	Neither
o:	ou	SDS	o:	FDS	o:	FDS	ou	Neither
ɛi	ɛi	SDS	ɛ:	FDS	ɛ:	FDS	ɛi	Neither
œy	œy	SDS	œ:	FDS	œ:	FDS	œy	Neither
ɑu	ɑu	SDS	ɑ:	FDS	ɑ:	FDS	ɑu	Neither
a:ʀ	a:ɹ	SDS	a:ʀ	FDS				
ɛ	ɛ	SDS+FDS	ɛ:	neither	ɛ	SDS+FDS	ɛ:	Neither
i	i	SDS+FDS	i	SDS+FDS	i	SDS+FDS	i	SDS+FDS
u	u	SDS+FDS	u	SDS+FDS	u	SDS+FDS	u	SDS+FDS
a:	a:	SDS+FDS	a:	SDS+FDS	a:	SDS+FDS	a:	SDS+FDS

**Figure 1:**
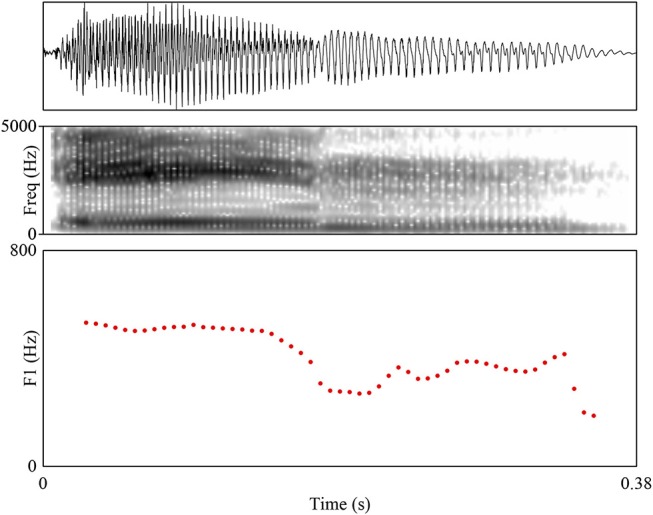
Example waveform, spectrogram, and F1 trajectory (the critical difference between diphthongal and monophthongal realizations) for the SDS realization of /e:n/ as [ein]. Toward the end of the vowel, the F1 falls.

**Figure 2:**
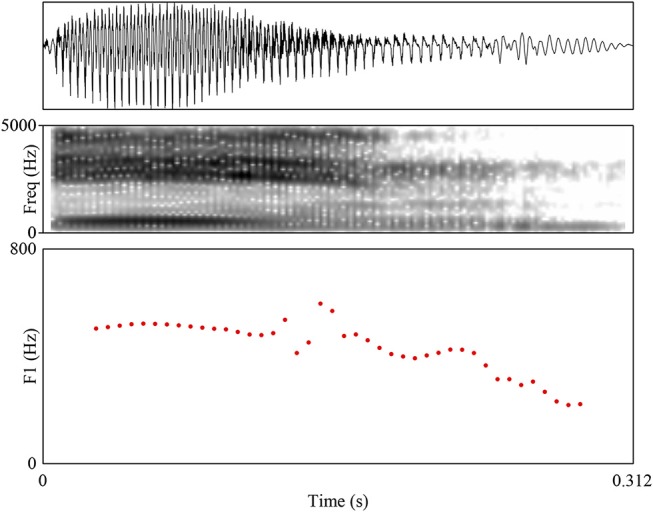
Example waveform, spectrogram, and F1 trajectory (the critical difference between diphthongal and monophthongal realizations) for the non-SDS realization of /e:n/ as [e:n]. The F1 stays stable throughout the vowel.

Figures 1 and 2 show spectrograms of the word /e:n/ realized as [ein] and [e:n] that demonstrate the difference under discussion. A crucial property of the experiment is that only the target phoneme (or phoneme sequence) was realized in a specific way; the remainder of the word was produced naturally. This prevents confounding the specific effect of allophone pronunciation with a global effect of regional accent, which is precisely the distinction that this study aims to tease apart. A reviewer additionally asks if there are orthographical differences between Standard Dutch and Flemish Dutch that could influence FDS' performance on the task; this is not the case.

Each prime was presented auditorily followed by a target presented visually. The targets were selected from the same set of 309 words as the primes. The pairing of targets to primes is as follows. In three conditions (19.5% of the experiment), viz. /e:,ɛi,a:ʀ/, the prime word and target word were the same; in the other conditions (80.5% of the experiment), the word on the screen was a random selection (without replacement) of the non-/e:,ɛi,a:ʀ/ words in the experiment.

### 3.3. Procedure and Data Acquisition

Participants were seated in front of a computer screen, loudspeakers, and a microphone, in a sound-attenuated and electrically-shielded booth. The experiment consisted of 618 trials with three breaks in between, spaced evenly throughout the experiment. All trials were presented on the computer using PsychoPy version 1.83.04. Before the experiment started, participants were presented instructions on the computer screen, which were also read aloud by a male Standard-Dutch speaker with a neutral (i.e., Randstad) accent.

**Figure 3:**
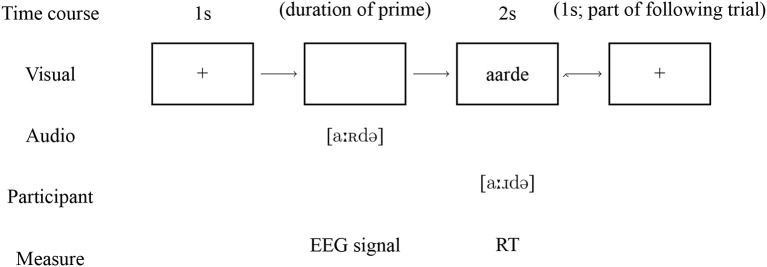
Example trial for the production task.

Each trial started with a black screen, followed by auditory presentation of the prime. When this prime had finished playing, the target word appeared on the screen (presented orthographically), which participants had been instructed to read aloud. Between two trials, a fixation cross was presented for 1 s. The data collected from the task are ERP responses to the prime words and vocal reaction times (i.e., speaking latencies) to the target words. A diagram showing an example trial and the data recorded from it is shown in Figure 3.

The manipulation took place in the primes: for each of the 309 words recorded, participants heard both the Standard-Dutch variant and, in a different trial, the non-Standard-Dutch variant. Which of these two variants was presented first for each word was randomized and counterbalanced.

During the whole task, continuous-time EEG activity was recorded using 32 Ag/AgCl electrodes with a sampling rate of 512 Hz. Two flat electrodes at the mastoids provided a reference signal, which was subtracted from the EEG signal in off-line processing; an additional four flat electrodes recorded horizontal and vertical extra-oculograms. In addition to the EEG activity, the speech of the participant was recorded while they realized the target word. Recording was started immediately after the prime word was presented, right when the target word became visible on the screen.

## 4. Results

### 4.1. Reaction Times

The words realized by the participants were aligned to their phonetic transcriptions (which were obtained from CELEX) using the Viterbi forced aligner present in HTK (Young et al., 2002). Forced alignment of speech sounds to phonemes is a more principled measure of speech onset time than thresholding raw acoustic energy, as the procedure uses speech-specific information in the signal and can hence do a better job at separating speech from background noise. For every word, the program produced a list of start and end points of the individual consonants and vowels present in the speech stream. Vocal RTs were obtained by extracting the time index of the first phoneme following the word-initial silence. RTs were obtained with a granularity of 10 ms.

The effects of the various factors in the design on these reaction times were analyzed by means of a generalized linear mixed-effects model with identity link and gamma errors^2^, following Lo and Andrews (2015). The fitting engine used for the model was function glmer from R (R Core Team, 2019) package lme4 (Bates et al., 2015). Fixed effects were added for “Group” (treatment-coded: 0 = SDS, 1 = FDS), “Allophone” (treatment-coded: 0 = non-Standard-Dutch; 1 = Standard-Dutch), “Session” (coded for linear and quadratic trends using orthogonal polynomials), “Identity” (treatment-coded: 1 = the prime and target word were the same, which was the case in the /a:ʀ,e:,ɛi/ conditions; 0 = the prime and target words differed), “Condition” (sum-coded), and all possible interactions. Using R package buildmer (Voeten, 2019), random slopes by participants and words were included over all terms as long as the model would still converge; these terms were entered in the order of their contribution to the log-likelihood, such that when the model eventually failed to converge, the most information-rich random slopes had been included. From this maximal model, terms were excluded in backward stepwise order based on the change in BIC. (Given the large number of interaction parameters present in the maximal model, BIC is a more natural elimination criterion than the likelihood-ratio test.) The raw data for these models are available in the Supplementary Materials as file RTdata.csv. The code used to fit the models is available in the Supplementary Materials as file RTcode.R.

Results of the analysis are shown in Table 3. Because the model used an identity link, the resulting model coefficients are directly interpretable as milliseconds of response latency. The intercept is placed at 827 ms ($\hat{\beta }$ = 827.16, *SE* = 2.22, *t* = 372.06, *p* < 0.001). This reflects the temporal onset of the first phoneme in the participant's response, for the Dutch control participants when they were presented with non-identity, Standard Dutch targets. Participants became slightly slower over the three sessions ($\hat{\beta }$ = 54.22, *SE* = 1.86, *t* = 29.13, *p* < 0.001), although the speed loss between sessions 2 and 3 was not as large as the speed loss between sessions 1 and 2 ($\hat{\beta }$ = –10.59, *SE* = 2.90, *t* = –3.65, *p* < 0.001). Overall, the FDS were slower than the SDS ($\hat{\beta }$ = 41.32, *SE* = 1.93, *t* = 21.37, *p* < 0.001). Identity primes incurred faster RTs than non-identity primes ($\hat{\beta }$ = –46.64, *SE* = 2.03, *t* = –23.02, *p* < 0.001), although this advantage was smaller for the FDS ($\hat{\beta }$ = 11.61, *SE* = 2.28, *t* = 5.10, *p* < 0.001). Overall, the non-Standard-Dutch allophones incurred slightly faster RTs than the Standard-Dutch allophones, although the size of this effect was smaller than the 10 ms granularity with which HTK had provided the reaction times ($\hat{\beta }$ = –6.00, *SE* = 1.67, *t* = –3.59, *p* < 0.001).

**Table 3:** Fixed-effect coefficients of the reaction-times analysis.

***Factor***	***Estimate (SE)***	***t***	***p***	***Sig*.**
(Intercept)	827.16 (2.22)	372.06	<0.001	***
Session (Linear)	54.22 (1.86)	29.13	<0.001	***
Session (Quadratic)	–10.59 (2.90)	–3.65	<0.001	***
Group = FDS	41.32 (1.93)	21.37	<0.001	***
Prime = Identity	–46.64 (2.03)	–23.02	<0.001	***
Allophone = Incorrect Dutch	–0.15 (1.71)	–0.09	0.93	
Group = FDS × Prime = Identity	11.61 (2.28)	5.10	<0.001	***
Prime = Identity × Allophone = Incorrect Dutch	–6.00 (1.67)	–3.59	<0.001	***

*The model additionally includes random intercepts by participants and by words, and random slopes for the factor “Session” by participants and by words*.

*** *p <0.001*.

### 4.2. ERP Results

**Figure 4:**
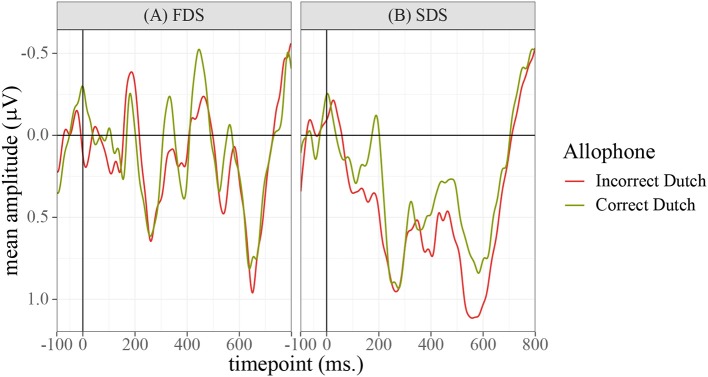
Grand-average waveforms calculated over the full dataset, averaged over all participants, electrodes, and the three sessions.

The ERP data were detrended and an off-line bandpass filter was applied passing a frequency domain of 1–30 Hz. Epochs were time-locked to the onset of the prime words and were extracted 0–800 ms post-stimulus-onset, after subtracting a –100 ms baseline^3^. Epochs contaminated by eyeblinks or other movement-related artifacts were rejected. The resulting grand-average waveforms (averaged over all participants, items, and electrodes), are shown in Figure 4.

**Figure 5:**
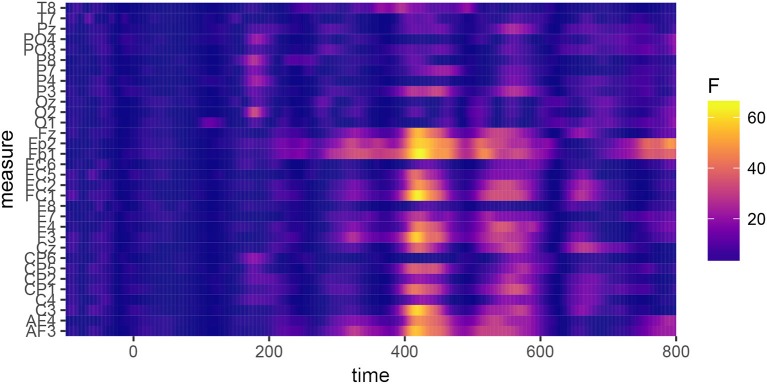
Permutation tests performed on the whole dataset, showing the effect of the factor “Group.” A significant difference can be observed, which reaches permutation-based significance between 390 and 470 ms.

**Figure 6:**
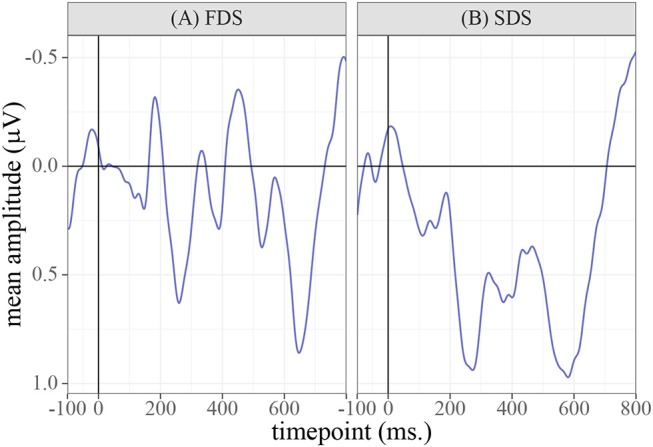
Grand-average waveforms calculated over the full dataset, averaged over the three sessions and the two allophone conditions. A difference in amplitude can be observed between the FDS and the controls, which reaches permutation-based significance in the 390–470 ms window.

**Figure 7:**
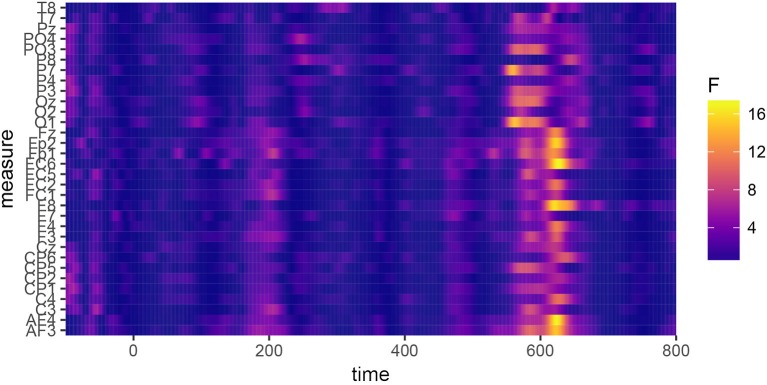
Permutation tests performed on the data for the [ɛ~ɛ:] contrast, showing the effect of the factor “Allophone×Group.” A significant difference can be observed, which reaches permutation-based significance between 560 and 660 ms.

**Figure 8:**
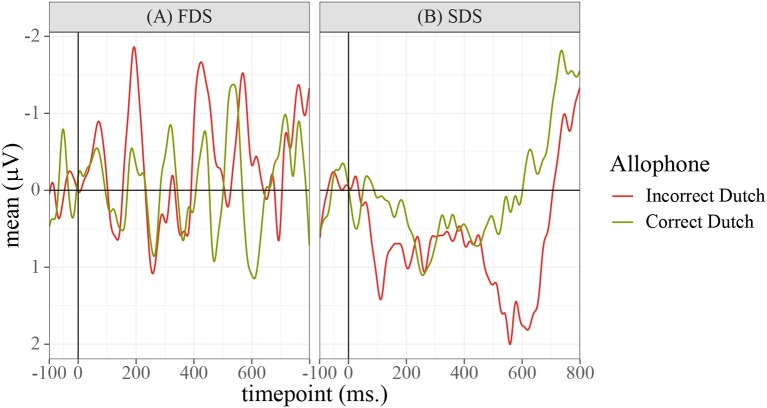
Grand-average waveforms calculated over the [ɛ~ɛ:] condition only, averaged over all participants, electrodes, and the three sessions. A difference in amplitude can be observed between the two allophone conditions, in the SDS group only, which reaches permutation-based significance in the 560–660 ms window.

In order to determine the precise temporal window and ROI to be used in the statistical analyses and data plots, permutation testing (Maris and Oostenveld, 2007) was applied to identify the locations of significant differences between the conditions. Repeated ANOVAs were run on each 〈timepoint,electrode〉 pair in the data using R package permutes (Voeten, 2018). The design of the test was a 2×2 ANOVA with fixed factors for “Allophone” (encoding the type of allophone presented to the participant), “Group” (FDS or SDS), and the interaction between the two. Because it was conceivable that the experimental items /e:,ø:,o:,ɛi,œy,ɑu/ and the control items /ɛ,a:ʀ/ might be differentially sensitive to the manipulation of the prime allophones, the permutation tests were run twice: once on the full dataset (to identify global differences between the groups of participants and allophones) and once for each of the eight conditions separately (to identify possible differences between the experimental items, the control vowel /ɛ/, and the control consonant condition /a:ʀ/). The analysis of the whole dataset, plotted in Figure 5, identified a global effect of “Group,” ranging from 390 to 470 ms at frontal, central, and parietal sites. Figure 6 shows the grand-average waveforms corresponding to this global difference between the groups. The analyses of the individual vowels failed to identify meaningful windows in the allophonic conditions [ei~e:, øy~ø:, ou~o:, ɛi~ɛ:, œy~œ:, ɑu~ɑ:, a:ɹ~a:ʀ], but for the [ɛ~ɛ:] condition, an effect of “Allophone × Group” was observed at essentially all electrode sites within a temporal window of 560 to 660 ms. This effect is plotted in Figure 7; Figure 8 shows the corresponding grand-average waveforms. Reasons why only this condition elicited a significant ERP are discussed in section 5.

The effects found in Figures 5 and 7 appear to correspond, respectively, to the classic N400 and P600 effects. To analyze the N400 effect, each response was averaged over the 390–470 ms window and over the frontal and central electrodes. The resulting data were analyzed by means of a mixed ANOVA with fixed effects for “Group”, “Allophone”, and “Session”, and a complete random-effects structure for participants and items using the same procedure as in section 4.1. Terms were selected for inclusion in the model by means of backward stepwise elimination, using the significance of the change in log-likelihood as the inclusion criterion. The resulting model is shown in Table 4; the raw data for this model are available in the Supplementary Materials as file N400data.csv. The code used to fit the models is available in the Supplementary Materials as file N400code.R.

**Table 4:** Fixed-effect coefficients for the N400 effect.

***Factor***	***Estimate (SE)***	***t***	***df***	***p***	***Sig*.**
Intercept	1.43 (0.74)	1.94	17.78	0.07	
Group = FDS	–0.89 (1.04)	–0.85	17.97	0.41	
Session (Linear)	0.45 (0.18)	2.55	12,650.91	0.01	*
Session (Quadratic)	0.79 (0.17)	4.55	12,672.79	<0.001	***
Group = FDS × Session (Linear)	–1.17 (0.29)	–3.97	12,250.75	<0.001	***
Group = FDS × Session (Quadratic)	0.21 (0.27)	0.78	12,665.72	0.44	

* *p < 0.05*,

*** *p < 0.001*.

The results show significant effects for “Session (Linear)” ($\hat{\beta }$ = 0.45, SE = 0.18, *t*_12, 650.91_ = 2.55, *p* = 0.01) and “Session (Quadratic)” ($\hat{\beta }$ = 0.79, SE = 0.17, *t*_12, 672.79_ = 4.55, *p* < 0.001). The linear component shows that the N400 became less pronounced over the three sessions, whereas the quadratic component implies that the N400 shrinkage between sessions 2 and 3 was larger than the reduction between sessions 1 and 2. The linear component additionally entered into a significant interaction with “Group = FDS” ($\hat{\beta }$ = −1.17, SE = 0.29, *t*_12, 250.75_ = −3.97, *p* < 0.001). This suggests that the linear trend of decreasing N400 amplitudes was significantly less pronounced for the FDS than it was for the SDS.

To analyze the P600 effect, the data were averaged over the 560–660 ms window and all electrodes. The data were analyzed in the same way as for the N400 effect. The model containing the terms that remained after stepwise elimination is shown in Table 5; the raw data for this model are available in the Supplementary Material as file P600data.csv. The code used to fit the models is available in the Supplementary Material as file P600code.R.

**Table 5:** Fixed-effect coefficients for the P600 effect.

***Factor***	***Estimate (SE)***	***t***	***df***	***p***	***Sig*.**
Intercept	0.81 (0.51)	1.60	24.60	0.12	
Session (Linear)	–0.76 (0.31)	–2.47	1,179.68	0.01	*
Session (Quadratic)	–0.17 (0.29)	–0.59	1,400.81	0.56	
Allophone = Non-SDS	1.70 (0.44)	3.86	1,394.70	<0.001	***
Group = FDS	0.49 (0.74)	0.66	27.79	0.51	
Allophone = Non-SDS × Group = FDS	–2.51 (0.67)	–3.76	1,395.54	<0.001	***

* *p < 0.05*,

*** *p < 0.001*.

The results show a significant effect for “Session (Linear)” ($\hat{\beta }$ = −0.76, SE = 0.31, *t*_1, 179.68_ = −2.47, *p* < 0.01). This suggests that the average amplitude in this window became slightly smaller in magnitude over the three sessions. A significant main effect was found for “Allophone = Non-SDS” ($\hat{\beta }$ = 1.70, SE = 0.44, *t*_1, 394.70_ = 3.86, *p* < 0.001), indicating that the non-SDS allophone elicited a much larger P600 response than the SDS allophone. However, this factor interacted significantly with “Group = FDS” ($\hat{\beta }$ = −2.51, SE = 0.67, *t*_1, 395.54_ = −3.76, *p* < 0.001), such that the P600 was completely negated in the FDS and in fact only showed up in the SDS group.

### 4.3. Topographical Distribution

**Figure 9:**
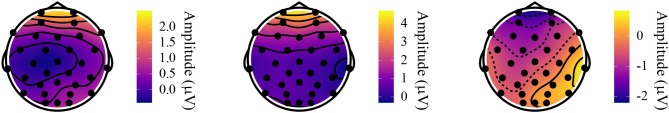
Topographical distribution of the N400 effect. The left head shows the FDS, the middle head shows the SDS, and the right head shows the difference between the two.

**Figure 10:**
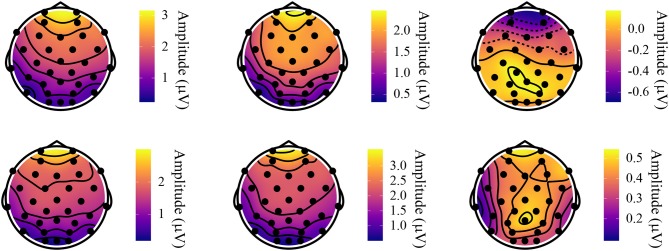
Topographical distribution of the P600 effect. The top three heads show the FDS, and the bottom three heads show the SDS. From left to right, both rows display, respectively: the SDS-allophone words; the Non-SDS-allophone words; the difference between the two.

The topographical distribution for the two effects is shown in Figure 9 for the N400 and Figure 10 for the P600 effect. For the N400, both the FDS and the SDS showed the lowest activity in central-parietal areas. The difference between the two was that the FDS's activity was lower than the SDS's at especially the frontal and frontal-central sites. Since this between-group difference was persistent throughout the whole experiment, it also shows up in the topographical plots of the P600 effect. In those plots, the FDS do not show any interpretable differences between the [ɛ] and the [ɛ:] allophones, other than the aforementioned frontal activity being more negative for the latter allophone. The SDS, however, show significantly more activity in parietal-occipital areas for the [ɛ:] allophone compared to the [ɛ] allophone, which corresponds to the classic ROI of the P600.

## 5. Discussion

The reaction-time data do not show any meaningful results for the research question. An expected effect of identity priming, with a plausible effect size of 47 ms facilitation, was found, but no significant difference in this facilitatory effect was found between the SDS and Non-SDS allophones. What was found, however, was that the FDS were in general slower responders than the SDS, by approximately 41 ms, an effect that is of similar magnitude to the identity-prime effect. This is in line with similar findings on accented-speech perception by Floccia et al. (2006, 2009).

One of the two main findings of this study is the difference in N400 amplitudes between the FDS and the SDS over the three sessions of the experiment. The magnitude of the N400 decreased in both groups over the three sessions, but did less so for the FDS than it did for the SDS, with an effect size of −1.17μV. The aforementioned findings by Dambacher et al. (2006) relating the N400 to general familiarity can explain this result: the FDS had less experience with Standard Dutch speech than the SDS did, and therefore were not as strongly facilitated in sessions 2 and 3 by their previous experiences with session 1. This result mirrors the behavioral findings by Floccia et al. (2009), who show that the processing impairment incurred by accented stimuli does not improve with more exposure. The present study extends this finding, by showing that it has an electrophysiological correlate in the N400.

The second main finding of this study was the P600 found when the phoneme /ɛ/ was realized as [ɛ:], which is an impossible realization of this phoneme (cf.Witteman et al., 2015). This phonological P600 is in line with recent papers, particularly those by Domahs et al. (2009) and Pater et al. (submitted). The effect was only found in the [ɛ~ɛ:] condition, which differed from the other conditions in one way, namely that the [ɛ:] is not just *phonologically* illegal, but also does not exist as an allophone of *any* phoneme in either SDS or FDS, making this condition most similar to Witteman et al. (2015). This sheds new light on the phonological P600 found by Domahs et al. (2009) and Pater et al. (submitted). They obtained P600s for allophonic violations, but their critical conditions were phonologically neutralizing. The artificial rule violated in Pater et al. (submitted) was a voicing-agreement rule between stop consonants; the Domahs et al. (2009) study investigated phonotactic violations by, again, stop consonants. In both studies, participants' native languages (English and German, respectively) contained a full stop system, making these specific violations cross phoneme boundaries. The present study's finding of the same P600 effect in the [ɛ~ɛ:] condition, but not the allophonic-violation conditions, implies that the phonological P600 is restricted to these neutralizing violations.

What remains to be discussed is the finding that the P600 was only observed in the SDS, and not in the FDS. The most likely explanation is that this is due to the FDS being less familiar with Standard Dutch, and therefore being less disturbed (or not significantly more than their baseline levels) by the [ɛ:] realizations. This interpretation is supported by the finding that their N400 amplitudes decreased less steeply over the course of the three sessions. Note however that, while it was smaller, the FDS group still showed a decrease in N400, just as the SDS group did. This suggests that long-term accommodation is possible, and that the FDS may simply require more exposure. The present study cannot shed any light on possible future long-term accommodation by the FDS to the Standard Dutch accent in general, because the present set-up cannot distinguish between accommodation to the differences in accent and accommodation to this specific experiment. Further research, with diverse stimuli over the multiple sessions, is necessary.

The differences between the SDS and the FDS have implications for our knowledge of the neural processing of on-going historical phonological change. The FDS, who serve as a proxy for a more conservative stage of Dutch, showed increased N400 amplitude by the three sessions and did not show significant P600 modulation in the condition where it was found for the SDS. These findings suggest that the present study successfully managed to elude the robust perceptual compensation mechanisms discussed in the Introduction. It additionally supports the logical assumption that this elusion is not permanent: while the FDS's N400s did not shrink as much as the SDS's did, they did shrink nonetheless. It is conceivable that eventually, the two groups' N400s would come to coincide, which may be the point at which an on-going change can be considered to have been acquired. The finding of the P600 in the [ɛ~ɛ:] condition for the SDS only further specifies the conditions under which this phonological P600 can be elicited. Of historical phonological change, this result implies that on-going sound change in the form of new allophonic variation is processed more subtly by the human perceptual apparatus than a phoneme merger or split.

The present study is not without its limitations. One difference between the manipulation in the present study vs. the manipulation used by Witteman et al. (2015) is that they used cross-spliced speech, while the present study used natural speech produced by a trained phonetician. A small but critical difference between the present study and Witteman et al. (2015) means that this study is not as critically reliant on splicing as theirs was. This is the fact that, in contrast to Witteman et al. (2015), the present study used entirely native Dutch material: even the [ɛ:] realizations correspond to perfectly fine Dutch phones, found in normal Dutch speech as the realizations of /ɛi/ before coda /l/. Nonetheless, the gain in naturalness of the speech material due to the absence of splicing artifacts might have been offset by a loss in naturalness of the manipulated stimuli realized by the speaker. While none of the participants commented on certain stimuli sounding artificial, and the speaker was phonetically trained and hence used to the task of realizing particular stimuli, this can be seen as a point of criticism in the design. In addition, the paradigm used in the present experiment—listening to single words and reading words aloud as a cover task—was one chosen out of convenience, this study being an exploratory part of a larger project for which this set-up was advantageous. Finally, the sample size—23 vs. 26 EEG recordings, but only 2 × 10 participants—in the present experiment was comparatively small. In sum, the effects found in this pilot experiment are in need of independent replication. I recommend that these findings be re-investigated in different languages using different paradigms, to ascertain whether the effects are cross-linguistic reflections of the processing of phonological differences between accentual varieties, or whether they are specific to these varieties of Dutch, or even to this specific task.

## 6. Conclusion

This study identified two electrophysiological correlates of accent processing and the processing of on-going phonological change in Standard Dutch and Flemish Dutch listeners to (experimentally-controlled) Standard Dutch speech. The amplitude of the N400, measuring listeners' familiarity with the general accent in which the experimental stimuli were spoken, was decreased for the Flemish Dutch group compared to the Standard Dutch group, indicating that they were more cautious in applying their predictive processing abilities to the unfamiliar accent (*pace* Goslin et al., 2012). This effect decreased over the three measurement sessions, indicating that over the course of nine months, the Flemish Dutch participants became more familiar with Standard Dutch speech, or at least the Standard Dutch speech of these experimental stimuli. In addition, a P600 effect was found for a very specific violation, viz. the realization of /ɛ/ as the illicit realization [ɛ:], in the Standard Dutch group of listeners only. This shows that the brain is capable of detecting this specific type of violations (viz. violations that cross a phoneme boundary, *pace* Witteman et al., 2015), but only after sufficient familiarity with the general accent is achieved (as implied by the significant difference in sensitivity to this violation between the Flemish Dutch group and the Standard Dutch group).

Inherent limitations to this exploratory study, particularly concerning the number of participants and the way in which the stimuli were created, mean that the results of this experiment need to be subjected to independent replication using different languages and paradigms before any definitive conclusions should be drawn. The present pilot experiment, however, has taken the first steps toward an electrophysiological investigation of the processing of on-going historical phonological change.

## Data Availability

The datasets analyzed for this study are available on request to the corresponding author. The datasets used in the statistical analyses are also included in the Supplementary Materials.

## Ethics Statement

This study was carried out in accordance with the recommendations of the Ethics Code for linguistic research in the faculty of Humanities at Leiden University with written informed consent from all subjects. All subjects gave written informed consent in accordance with the Declaration of Helsinki. The protocol was approved by the Leiden University Center for Linguistics.

## Author Contributions

CV and CL conceived the experiment, designed the stimuli and edited the manuscript, CV performed the experiments, analyzed the data and wrote the manuscript.

### Conflict of Interest Statement

The author declares that the research was conducted in the absence of any commercial or financial relationships that could be construed as a potential conflict of interest.

## Footnotes

^1^Unstressed vowels in Dutch may optionally undergo reduction (Booij, 1995), which could result in the elision of the crucial upgliding diphthongization.

^2^Inverse-Gaussian (Wald) errors were also considered, but provided a worse fit to the data (higher AIC) compared to the gamma-errors model.

^3^The rather short span of this baseline was necessary due to divergence of the baselines earlier than –100 ms.
